# Development and Usability Testing of a Computer-Tailored Decision Support Tool for Lung Cancer Screening: Study Protocol

**DOI:** 10.2196/resprot.8694

**Published:** 2017-11-16

**Authors:** Lisa Carter-Harris, Robert Skipworth Comer, Anurag Goyal, Emilee Christine Vode, Nasser Hanna, DuyKhanh Ceppa, Susan M Rawl

**Affiliations:** ^1^ Science of Nursing Care Department Indiana University School of Nursing Indiana University–Purdue University at Indianapolis Indianapolis, IN United States; ^2^ Cancer Prevention & Control Program Indiana University Melvin and Bren Simon Cancer Center Indianapolis, IN United States; ^3^ Indiana University School of Informatics and Computing Indiana University–Purdue University at Indianapolis Indianapolis, IN United States; ^4^ Indiana University School of Medicine Indianapolis, IN United States; ^5^ Indiana University Melvin and Bren Simon Cancer Center Indianapolis, IN United States

**Keywords:** lung cancer screening, informed decision making, shared decision making, patient decision aid, patient education, early detection of cancer, lung neoplasms, decision support techniques, decision making, computer-assisted

## Abstract

**Background:**

Awareness of lung cancer screening remains low in the screening-eligible population, and when patients visit their clinician never having heard of lung cancer screening, engaging in shared decision making to arrive at an informed decision can be a challenge. Therefore, methods to effectively support both patients and clinicians to engage in these important discussions are essential. To facilitate shared decision making about lung cancer screening, effective methods to prepare patients to have these important discussions with their clinician are needed.

**Objective:**

Our objective is to develop a computer-tailored decision support tool that meets the certification criteria of the International Patient Decision Aid Standards instrument version 4.0 that will support shared decision making in lung cancer screening decisions.

**Methods:**

Using a 3-phase process, we will develop and test a prototype of a computer-tailored decision support tool in a sample of lung cancer screening-eligible individuals. In phase I, we assembled a community advisory board comprising 10 screening-eligible individuals to develop the prototype. In phase II, we recruited a sample of 13 screening-eligible individuals to test the prototype for usability, acceptability, and satisfaction. In phase III, we are conducting a pilot randomized controlled trial (RCT) with 60 screening-eligible participants who have never been screened for lung cancer. Outcomes tested include lung cancer and screening knowledge, lung cancer screening health beliefs (perceived risk, perceived benefits, perceived barriers, and self-efficacy), perception of being prepared to engage in a patient-clinician discussion about lung cancer screening, occurrence of a patient-clinician discussion about lung cancer screening, and stage of adoption for lung cancer screening.

**Results:**

Phases I and II are complete. Phase III is underway. As of July 15, 2017, 60 participants have been enrolled into the study, and have completed the baseline survey, intervention, and first follow-up survey. We expect to have results by December 31, 2017 and to have data analysis completed by March 1, 2018.

**Conclusions:**

Results from usability testing indicate that the computer-tailored decision support tool is easy to use, is helpful, and provides a satisfactory experience for the user. At the conclusion of phase III (pilot RCT), we will have preliminary effect sizes to inform a future fully powered RCT on changes in (1) knowledge about lung cancer and screening, (2) perceived risk of lung cancer, (3) perceived benefits of lung cancer screening, (4) perceived barriers to lung cancer screening, (5) self-efficacy for lung cancer screening, and (6) perceptions of being adequately prepared to engage in a discussion with their clinician about lung cancer screening.

## Introduction

Lung cancer screening is a US Preventive Services Task Force (USPSTF) grade B recommendation, indicating there is high certainty that the overall benefits are substantial [1]. The National Lung Screening Trial, on which the recommendation is based, found a 20% lung cancer-related mortality reduction for long-term smokers screened annually with low-dose computed tomography (LDCT) compared with chest radiography [2]. However, lung cancer screening with LDCT is a complex issue. Screening has associated risks and potential harms that complicate the decision to screen [1]. Most notable among these are false-positive results and incidental findings, which can lead to a cascade of unnecessary invasive testing [1,2]. Therefore, the USPSTF recommends that the decision to screen for lung cancer should be the result of a shared decision-making process between a patient and their clinician. In addition to shared decision making being incorporated into the lung cancer screening guideline [1], for the first time, it is a requirement for reimbursement of a cancer screening test from Medicare [3].

Our team’s preliminary work revealed that most individuals eligible for lung cancer screening are unaware of, or confused or misinformed about (1) how lung cancer screening is performed, (2) the benefits and associated risks of screening, and (3) the causes of and associated risk factors for developing lung cancer [4]. It is critical to increase long-term smokers’ awareness of and education about lung cancer and screening. Education is an essential component of the shared decision-making process, and the USPSTF guidelines provide criteria for whom to engage in shared decision making about lung cancer screening [1]. The screening decision should, therefore, be the result of a clinical encounter in which the clinician and patient engage in shared decision making. Patients who are involved in decision making about their health have better outcomes [5], and shared decision making is ideally suited for the complex nature of the decision to screen, or not, for lung cancer. However, awareness of lung cancer screening remains low in the screening-eligible population [6]. In our team’s recent study exploring the decision to opt in or out of lung cancer screening, our findings highlighted the prevalence of time-constrained clinical encounters and their negative effect on the shared decision-making process about screening [7]. When a patient visits their clinician and has never heard of lung cancer screening before, engaging in the shared decision-making process to arrive at an informed decision can be a challenge. Methods to effectively support both patients and clinicians to engage in shared decision making are essential. Importantly, to facilitate this process, effective methods to prepare patients to *have* these important discussions with their clinician are needed.

Several decision aids [8-11] have been developed in response to the USPSTF lung cancer screening guideline and Medicare mandate for shared decision making. However, few are theoretically grounded. To our knowledge, 2 theoretically grounded lung cancer screening decision aids have been published in the literature and both are conceptually framed from a perspective of risk [8,9]. Volk and colleagues developed the video *Lung Cancer Screening: Is It Right for Me?* [8]. This 6-minute video provides information about risk factors for lung cancer, and harms and benefits of lung cancer screening, and presents vignettes depicting trade-offs between harms and benefits to clarify values [8]. Initial feasibility showed that this decision aid increased knowledge (*P*<.01) and supported readiness to make a decision to screen for lung cancer as reflected in significantly higher values clarity scores [8]. Lau and colleagues developed a Web-based decision aid guided by the Ottawa Decision Support Framework using an established prediction model to compute baseline lung cancer risk and an individual’s chance of benefiting from, and risk of being harmed by, screening [9]. Knowledge of lung cancer and screening increased (*P*<.001) and decisional conflict decreased (*P*<.001) in initial feasibility testing [9]. Other commercially developed lung cancer screening decision aids focus on calculating personal risk for the development of lung cancer with subsequent screening recommendations based on the calculated risk [10,11].

As mentioned, lung cancer screening is a complex issue with associated risks and potential harms that complicate the decision to screen. During phase I with our community advisory board (CAB; described in more detail below), participants expressed concern about messaging for former smokers being similar to that for current smokers and the potential for increased perceived stigma. This highlighted the importance of tailoring a decision support tool based on smoking status. With careful consideration of the messaging, we chose a tailored approach because tailored interventions are more effective than nontailored ones, and have been shown to improve knowledge, change health beliefs, and promote health behavior change in other types of cancer screening such as breast and colorectal cancers [12-17]. Our previous research revealed that perceived risk was not associated with actual screening behavior in lung cancer [18]. Regardless, it is important for any decision support tool in lung cancer screening to include risks for the development of lung cancer; however, to support the shared decision-making process in lung cancer screening, it is critical that lung cancer screening decision aids go beyond assessing risk and that they tailor messages based on multiple salient variables that may be personally relevant to the individual. Lung cancer screening decision aids should also leverage the previsit time frame (ie, during either the time spent waiting in the clinic before the clinician comes in for the visit or the week leading up to a scheduled well-care appointment, in which patient education can be consumed at home) to prime patients with new knowledge about lung health and the option of screening. Educating patients during the previsit time period has the potential to enhance the subsequent clinical encounter for patient engagement in a shared decision-making process.

It is also important to acknowledge the role stigma may play in lung cancer screening discussions and decisions. Individuals qualify for lung cancer screening based on their history of long-term tobacco use, and smokers are different from populations targeted for other types of cancer screening, which base eligibility on age and sex. Smokers have reported perceiving blame and feeling stigmatized in clinical encounters secondary to their status as a current or former smoker [4,19]. Further, initial focus group discussions with screening-eligible individuals revealed that former smokers did not wish to be addressed as a current smoker. Therefore, patient decision aids for lung cancer screening may benefit from tailoring messages and content by smoking status.

This paper discusses the development and usability testing of a computer-tailored decision support tool called LungTalk. This decision support tool was developed using the USPSTF lung cancer screening guideline and the qualifying and certification criteria of the International Patient Decision Aid Standards instrument version 4.0 as a guide [20]. The purpose of LungTalk is to prepare individuals for the shared decision-making process about lung cancer screening by educating individuals about (1) lung health broadly including the effects of nicotine, (2) risk factors for the development of lung cancer, (3) the option of lung cancer screening with LDCT of the chest, and (4) risks and benefits of lung cancer screening. The content and messages of LungTalk are tailored by smoking status. LungTalk is Web based and can be delivered via email or sent to a patient via a health system’s patient portal with an embedded weblink prior to an upcoming clinic visit. LungTalk can also be delivered via a tablet-based device in the clinic.

## Methods

We developed a prototype of a computer-tailored decision support tool using user-centered design based on the most recent USPSTF lung cancer screening guidelines and the International Patient Decision Aid Standards instrument version 4.0 checklist. Its aim was to (1) educate individuals about lung health and lung cancer screening, (2) prepare them to engage in a discussion about screening with their clinician, and (3) enhance the shared decision-making process between clinicians and screening-eligible patients. This study was approved by the Institutional Review Board at Indiana University prior to recruitment, and we obtained informed consent prior to study participation. We used a 3-phase process to develop and test the prototype in the target population. We describe the process below by phase.

### Phase I: Development

#### Overview

The computer-tailored decision support tool was developed by a team of researchers and clinicians with expertise in behavioral science, nursing, primary care, oncology, lung cancer screening, and informatics. Informed by a CAB comprising 10 screening-eligible individuals, the computer program was named LungTalk because the program goes beyond educating individuals solely about lung cancer screening to also educate broadly about lung health. We designed LungTalk to increase the users’ awareness of potential risks related to long-term nicotine exposure, benefits of and potential harms related to lung cancer screening, and importance of shared decision making in the decision to screen for lung cancer or not, and to prompt users to initiate a discussion with a clinician about lung cancer screening.

LungTalk is unique in that it tailors messages based on smoking status and provides a tailored printout to help patients initiate a discussion with their clinician about their lung health and the option of screening. Our prior qualitative research with screening-eligible individuals revealed that many former smokers perceived stigma in clinical encounters where their long-term tobacco use was a focus [4,7]. This highlighted the importance of considering the framing of messaging based on smoking status in the development of the content of the decision support tool, as well as the importance of providing support for a discussion through a tailored printout.

#### Recruitment of Community Advisory Board Members

Our target population for LungTalk was lung cancer screening-eligible individuals based on the USPSTF lung cancer screening criteria [1]. These included persons who are aged 55 to 80 years, and current smokers or former smokers who had quit within the past 15 years with a minimum 30-pack-year tobacco smoking history [1]. We recruited CAB members from the local community using newspaper advertisement and through the Indiana University Health Lung Screening Clinic, Indianapolis, Indiana, USA, to ensure equal representation of individuals who had recently been screened for lung cancer and individuals who had not been screened.

#### Development of LungTalk With the Community Advisory Board

In phase I, we assembled the CAB to provide critical input and feedback during the initial development of LungTalk. We held 3 CAB meetings over the course of 6 months under the direction of 2 researchers (LCH and SMR) to discuss individual components of the computer program. Guided by user-centered design and in consultation with the study’s design team (consisting of experts in informatics, user experience design and engineering, and visual communication), we asked members of the CAB to provide iterative feedback on the design and prototypes, preferences for how the program should “look and feel,” and expectations of how the program should work when used in real-world settings. More specifically, the CAB provided feedback on what should and should not be included; development of, and specific wording for, messages to constitute the content library for LungTalk; specific graphic components of the program; and the tailored printout. Each CAB meeting was audiotaped in order to accurately capture feedback from CAB members and facilitate prototype revisions with the design team. Digital audio recordings and field notes from the CAB meetings were reviewed by a researcher (LCH) and summarized for the design team.

### Description of LungTalk

LungTalk is a computer-tailored decision support tool that is theoretically grounded in the conceptual model on lung cancer screening participation [21]. This model links the health belief model to the precaution adoption process model and includes key psychological variables (eg, stigma, mistrust, fatalism, fear, and worry) as factors that may influence an individual’s decision to screen, or not, for lung cancer [21]. The LungTalk prototype is an interactive program that includes audio, video, and animation segments with tailoring algorithms for scripts presented from the master content library. In addition, LungTalk offers the option of saving or printing a tailored printout at the end. This printout highlights key points related to lung health and screening tailored by smoking status, offers question prompts the user can use to initiate the discussion with their clinician, and tailors messages based on questions that remain important to the user that they wish to discuss further with their clinician. Literacy level has been considered in the development of LungTalk and messaging is presented at an eighth-grade level. In addition, in consideration of different ways people like to learn, the content is presented via narration as well as key text on screen.

### Phase II: Usability Testing

#### Overview

Following the initial prototype development of LungTalk, we conducted usability testing with 13 screening-eligible individuals (different from those constituting the CAB). Table 1 presents the sociodemographic characteristics of the CAB and usability testers.

#### Data Collection

Pretesting took place in the usability testing laboratory at the Indiana University School of Informatics. We used the method for iterative usability evaluation based on the Milano-Lugano evaluation method systematic usability inspection technique [22]. This method enabled us to identify communication breakdowns and recommend design improvements on task support, information architecture, navigation design, and interaction mechanisms.

**Table 1:** Phase I and phase II participant sociodemographic and health status characteristics.

Characteristics		Community advisory board (n=10)	Usability testing (n=13)
Age (years), mean (SD)		63.3 (6.7)	62.6 (7.2)
**Sex, n (%)**			
	Male	6 (60)	10 (77)
	Female	4 (40)	3 (23)
**Race, n (%)**			
	White	4 (40)	10 (77)
	Black	6 (60)	3 (23)
**Education, n (%)**			
	Less than high school	1 (10)	1 (8)
	High school graduate	4 (40)	0 (0)
	Some college	4 (40)	7 (54)
	College graduate or higher	1 (10)	5 (39)
**Income (US $), n (%)**			
	<25,000	2 (20)	6 (46)
	25,000-50,000	5 (50)	2 (15)
	>50,000	3 (30)	5 (39)
**Health insurance, n (%)**			
	Government	6 (60)	5 (39)
	Private	4 (40)	8 (62)
**Smoking status, n (%)**			
	Current smoker	5 (50)	4 (31)
	Former smoker	5 (50)	9 (69)
**Family history of lung cancer, n (%)**			
	Yes	4 (40)	5 (39)
	No	6 (60)	8 (62)

Each usability testing session was facilitated by the researcher and a member of the study design team. The primary purpose of the usability testing session was to identify any programming errors or design issues that would prevent a satisfactory user experience and to curate additional feedback on the overall program.

Usability testing involved the participant using the program twice. First, the participant used the program without interruption. The researcher and study design team member observed how the participant interacted with the program and completed an investigator-developed observer checklist (see Multimedia Appendix 1). The participant was then asked to use the program a second time and was stopped at key points for the researcher to ask questions. The assessment included questions about specific content, messaging, points of potential confusion, opinions on visual, written, and verbal content, and flow design. On completion, the participant completed 2 questionnaires: (1) the 10-item System Usability Scale (SUS; see Multimedia Appendix 2); and (2) the 21-item Acceptability and Satisfaction Questionnaire (see Multimedia Appendix 3) [23,24].

#### Measures

We measured usability with the 10-item SUS. The SUS comprises 4-point Likert-response option items (1=strongly disagree, 2=disagree, 3=agree, 4=strongly agree). Participants rated items across a variety of specific tasks, including ease of use, consistency of the computer program, perception of how integrated the program felt during use, and perception of how well the computer program was able to prepare the user to discuss lung cancer screening with their clinician. In addition, open-ended questions were provided to allow participants to give feedback on negative and positive impressions of the overall program, as well as specific components. Participants were also asked to provide an overall letter grade rating for the program (ranging from A=excellent to F=unacceptable).

We measured acceptability and satisfaction with a 22-item questionnaire using a 4-point Likert-response option (1=strongly disagree to 4=strongly agree). In addition to overall satisfaction with the computer program, items assessed a variety of acceptability- and satisfaction-related components of the computer program, such as (1) amount of time to complete, (2) clarity of the messages, (3) enjoyment with use, (4) content relatability, and (5) ability to engage the user.

#### Analysis and Results

All 13 participants viewed LungTalk twice as previously described. None of the participants experienced any technical difficulties during testing (eg, interruption of Internet service, computer program pausing or ending unexpectedly). However, 3 participants had difficulty recognizing the forward arrow button at the bottom of the screen to advance the program at the beginning. All 3 recommended that this button either be highlighted in green or flashing to indicate that the user needs to click the button to advance the program. Many users also recommended changing the settings of the program to autoadvance forward through the material in different sections to eliminate the need to click a button. We examined usability with the SUS. Reverse-coded items on the SUS were transformed for analysis, and total SUS scores ranged from 62.5 to 85 on a 100-point scale with an overall mean of 75.8 (SD 7.9). Total scores on the Acceptability and Satisfaction Questionnaire ranged from 79.8 to 97.6 on a 100-point scale with an overall mean of 90.2 (SD 6.3). Slightly more than half of the participants gave LungTalk an overall A rating (ie, excellent; n=7, 54%) with the remainder giving LungTalk a B rating (good; n=6, 46%).

### Phase III: Community-Based, Web-Based Pilot Randomized Controlled Trial

#### Overview

Following development and usability testing of LungTalk, we are conducting a pilot randomized controlled trial (RCT) with the goal of obtaining preliminary effect size data for a future fully powered RCT. The pilot RCT will estimate the effect sizes of LungTalk on (1) changes in knowledge, (2) changes in health beliefs, and (3) participant perceptions of being adequately prepared to engage in a discussion with their clinician about lung cancer screening. Since the purpose of LungTalk is to prepare individuals for the shared decision-making process about lung cancer screening, the occurrence of a patient-clinician discussion about lung cancer screening and actual lung cancer screening completion are not the primary focuses in this initial study. However, for exploratory purposes, we will estimate the effect sizes of LungTalk for both.

#### Data Collection

Based on the overall objective of the pilot study to obtain preliminary effect size data, we need at least 12 participants per group to obtain reasonable effect size estimates to design a larger, well-powered trial [25,26]. To provide for the potential of attrition, 60 lung cancer screening-eligible participants who have not been screened for lung cancer have been recruited from the community using Facebook targeted advertisement [27]. We have randomly assigned participants to 1 of 2 groups after baseline data collection. The intervention group received LungTalk, and the enhanced control group received a nontailored lung screening information sheet compiled using patient education material from the American Cancer Society website [28]. Participants were randomly assigned and stratified by sex to each group. Stratified random assignment will ensure that the 2 groups are comparable in distribution. We are collecting data via REDCap (REDCap Consortium) at 3 time points via telephone: (1) baseline at recruitment, (2) within 1 week of completing the intervention, and (3) 3 months postintervention. REDCap is a secure Web-based app for building and managing online surveys and databases. REDCap provides audit trails for tracking data manipulation and user activity, as well as automated export procedures for secure data downloads to common statistical packages [29].

We are administering a baseline survey to collect data on sociodemographic and health status characteristics, lung cancer and screening knowledge, health beliefs (perceived risk of lung cancer, and perceived benefits of, perceived barriers to, and self-efficacy for lung cancer screening) [18], self-report of perception of preparation to engage in a patient-clinician discussion about lung cancer screening, and stage of adoption for lung cancer screening. We are measuring stage of adoption using an algorithm that is theoretically based on the precaution adoption process model [30]. This model categorizes individuals into 1 of 7 stages: unaware, aware but unengaged, undecided, decided not to act, decided to act, action, and maintenance [30]. At the completion of the baseline survey, each participant has been randomly assigned to either the intervention group or the enhanced control group. A link to either LungTalk or the lung screening information sheet was emailed to the participant based on their random assignment.

We administered a follow-up survey by telephone within 1 week of intervention completion. The survey included items to assess lung cancer and screening knowledge, health beliefs (perceived risk, perceived benefits, perceived barriers, and self-efficacy) [18], satisfaction with the intervention, self-report of perception of preparation to engage in a patient-clinician discussion about lung cancer screening, self-report of the occurrence of a patient-clinician discussion about lung cancer screening, and stage of adoption for lung cancer screening [30].

We will administer a second follow-up telephone survey 3 months after completion of the intervention. The 3-month follow-up survey will include items to assess self-report of the occurrence of a patient-clinician discussion about lung cancer screening, clinician recommendation, and stage of adoption for lung cancer screening [30]. For individuals who self-report completing lung cancer screening, we will verify the screening by mailing an authorization form to the participant to be signed and mailed back to the research office. A trained research assistant will verify the LDCT scan to screen for lung cancer using the information and signed authorization form by contacting the facility to request confirmation.

#### Analysis

We will compile deidentified data collected via REDCap and export it into a SAS file (version 9.4; SAS Institute). Data completeness will be assessed through descriptive analyses. Means and standard deviations or frequency distributions will be examined to check for coding errors and out-of-range values. All variables will be described with summary statistics appropriate for measurement level. Key analyses will be descriptive; we will calculate means and standard deviations and Cohen *d* effect sizes of study variables by group and for each study time point. We will calculate 95% CIs for the effect sizes using the nonparametric bootstrap approach based on 2000 bootstrap replications. For exploratory purposes, we will fit linear mixed-effects models with smoking status and race as factors, and a participant-specific random intercept to account for the association between the observations of the same participant. We will also evaluate the feasibility of study procedures. Therefore, we will calculate participation rates and rates of completion and retention of participants at (1) baseline survey, (2) 1-week postintervention survey, and (3) 3-month postintervention survey. For each, we will calculate the proportion of people who were recruited initially and retained at each stage, along with the associated 95% CI. Patterns of missing values will be examined and evaluated for randomness using the method described by Enders [31]. We will evaluate diagnostic plots and inferential tests for tenability of assumptions and apply appropriate remedial methods where required.

For the pilot RCT, we will estimate initial differences between the 2 intervention groups with respect to key study variables. We will recruit 30 participants per group to estimate effect sizes of LungTalk on (1) changes in knowledge, (2) changes in health beliefs, (3) self-report of participant perceptions of being adequately prepared to engage in a discussion with their clinician about lung cancer screening, (4) occurrence of a patient-clinician discussion about lung cancer screening, and (5) stage of adoption for lung cancer screening. Key analyses will be descriptive; we will calculate means and standard deviations of study variables by group. For exploratory purposes, we will also fit 2-way analysis of variance models with smoking status and race as factors.

## Results

We are conducting the pilot RCT. Recruitment began on June 15, 2017 using Facebook targeted advertisement. As of July 15, 2017, all 60 participants have been enrolled into the study and have completed the baseline survey, the intervention, and the first follow-up survey. We expect to have final results by December 31, 2017 and to have completed data analysis by March 1, 2018.

## Discussion

Results from pretesting LungTalk in the usability laboratory indicate that the computer program is easy to use, is helpful, and provides a satisfactory experience for the user. The pilot RCT will provide preliminary effect sizes of changes in (1) knowledge about lung cancer and screening, (2) perceived risk of lung cancer, (3) perceived benefits of lung cancer screening, (4) perceived barriers to lung cancer screening, (5) self-efficacy for lung cancer screening, and (6) participant perceptions of being adequately prepared to engage in a discussion with their clinician about lung cancer screening. We anticipate that LungTalk will be helpful to screening-eligible individuals as a tool to support those considering the option to screen, or not, for lung cancer. Specifically, LungTalk can help enhance the shared decision-making process in lung cancer screening by priming individuals with essential baseline knowledge for the discussion and decision-making process.

## Appendix

Multimedia Appendix 1 Observer checklist.

Multimedia Appendix 2 System Usability Scale.

Multimedia Appendix 3 Acceptability and Satisfaction Questionnaire.

## Abbreviations

CAB: community advisory board
LDCT: low-dose computed tomography
RCT: randomized controlled trial
SUS: System Usability Scale
USPSTF: US Preventive Services Task Force
