# Supplementary material for: Development and Usability Testing of a Computer-Tailored Decision Support Tool for Lung Cancer Screening: Study Protocol
Source: JMIR Res Protoc. 2017 Nov 16;6(11):e225. doi: 10.2196/resprot.8694 (PMC5709657; doi:10.2196/resprot.8694)
Supplement: Multimedia Appendix 1 [file resprot_v6i11e225_app1.pdf]

Observer Checklist

Instructions: Explain to the participant that you will observe them as they use the program from start to finish. *Note: Make a note of any problems the user has with the program (i.e., pausing for a few seconds when trying to complete a task, stumbling and having to backtrack in their steps or has to undo an action, expressions of audible frustration, taking a long time to complete a task)*.

1. Program starts without any problems. Yes No
2. User navigates easily through computer program. Yes No
    a. If not, please describe problems encountered:

____

____

____

3. Keeps user attention throughout. Yes No
4. Does the user encounter any errors while using the computer program? Yes No
    a. If yes, please describe:

____

____

____

5. Does the user appear frustrated with the program? Yes No

   If Yes, please circle:
    a. Slightly Frustrated
    b. Somewhat Frustrated
    c. Very Frustrated

6. Does the user appear nervous using the program? Yes No

   If Yes, please circle:

   a. Slightly Nervous
   b. Somewhat Nervous
   c. Very Nervous

7. Does the user appear hesitant using the program? Yes No

   If Yes, please circle:

   a. Slightly Hesitant
   b. Somewhat Hesitant
   c. Very Hesitant

8. Does the user appear to enjoy using the program? Yes No

9. Does the user appear to be having fun using the program? Yes No

Additional Comments (unsolicited from the user): ____

____

____

____

____

____

____
