# Supplementary material for: Development and Usability Testing of a Computer-Tailored Decision Support Tool for Lung Cancer Screening: Study Protocol
Source: JMIR Res Protoc. 2017 Nov 16;6(11):e225. doi: 10.2196/resprot.8694 (PMC5709657; doi:10.2196/resprot.8694)
Supplement: Multimedia Appendix 2 [file resprot_v6i11e225_app2.pdf]

Participant ID # _______________ System Usability Scale (SUS)

Instructions: Please check (√) the one answer for each item that is closest to your feelings.

		Strongly Disagree	Disagree	Agree	Strongly Agree
1	I think that I would like to use the program to help prepare me to have a discussion with my healthcare provider about lung screening.	1	2	3	4
2	I found the program unnecessarily complex.	1	2	3	4
3	I thought the program was easy to use.	1	2	3	4
4	I think that I would need the support of a technical person to be able to use this program.	1	2	3	4
5	I found the various functions in this program were well integrated.	1	2	3	4
6	I thought there was too much inconsistency in this program.	1	2	3	4
7	I would imagine that most people would learn to use this program very quickly.	1	2	3	4
8	I found the program very cumbersome to use.	1	2	3	4
9	I felt very confident using the program.	1	2	3	4
10	I needed to learn a lot of things before I could get going with this program.	1	2	3	4

Report Card

Instructions: Circle the letter that best describes the overall grade you would give to the computer program.

A = Excellent
B = Good
C = Average
D = Below Average
F = Unacceptable

Grade

A B C D F

Thank You!

Please return the questionnaire to the Researcher at this time
