# Supplementary material for: Development and Usability Testing of a Computer-Tailored Decision Support Tool for Lung Cancer Screening: Study Protocol
Source: JMIR Res Protoc. 2017 Nov 16;6(11):e225. doi: 10.2196/resprot.8694 (PMC5709657; doi:10.2196/resprot.8694)
Supplement: Multimedia Appendix 3 [file resprot_v6i11e225_app3.pdf]

Instructions: Please circle the number that best describes how much you agree or disagree with each statement.

		Strongly Disagree	Disagree	Agree	Strongly Agree
1	I could understand the messages I heard in the program.	1	2	3	4
2	The program took too much time.	1	2	3	4
3	Using the program made me nervous.	1	2	3	4
4	I enjoyed using the program.	1	2	3	4
5	The information I received was important to me.	1	2	3	4
6	I was very interested in the information from the program.	1	2	3	4
7	The program made me think about having a discussion with my provider about lung screening.	1	2	3	4
8	The messages in the program made sense to me.	1	2	3	4
9	The information in the program doesn't relate to me.	1	2	3	4
10	The information in the program was interesting.	1	2	3	4
11	Since using the program, I feel prepared to have a discussion about lung screening with my healthcare provider.	1	2	3	4

(continued)

		Strongly Disagree	Disagree	Agree	Strongly Agree
12	Time passed quickly when I completed the computer program.	1	2	3	4
13	I listened carefully to the messages in the program.	1	2	3	4
14	The information in the program was easy to understand.	1	2	3	4
15	The program seemed like it was meant just for me.	1	2	3	4
16	I would like to learn more about lung screening.	1	2	3	4
17	I don’t really need the information in the program.	1	2	3	4
18	I had trouble paying attention to the program.	1	2	3	4
19	A program like this could help me talk with my doctor about lung screening.	1	2	3	4

(continued)

		Strongly Disagree	Disagree	Agree	Strongly Agree
20	I would recommend this program to other people.	1	2	3	4
21	I did not like using the headphones with the program. (Write in N/A if not used)	1	2	3	4
22	Overall, I am very satisfied with the program.	1	2	3	4

Thank You!

Please return the questionnaire at this time
